# Association of donor heavy alcohol use with graft failure after deceased-donor liver transplantation stratified by donor sex and macrosteatosis in the OPTN/UNOS registry

**DOI:** 10.1038/s41598-026-48596-y

**Published:** 2026-04-15

**Authors:** Sangbin Han, Vatche G. Agopian, Justin A. Steggerda, Juhee Lee, Myungsuk Kim, Irene K. Kim, Ji-Hye Kwon, Alison Sanford, Yi-Te Lee, Ju-Dong Yang

**Affiliations:** 1 Department of Anesthesiology and Pain Medicine, Samsung Medical Center, Sungkyunkwan University School of Medicine, Seoul, 06351 Korea; 2 Department of Surgery, David Geffen School of Medicine at UCLA, Los Angeles, CA USA; 3 Department of Surgery, Cedars-Sinai Medical Center, Los Angeles, CA USA; 4 Samsung Medical Center, Sungkyunkwan University School of Medicine, Seoul, Korea; 5 Division of Gastroenterology and Hepatology, Cedars-Sinai Medical Center, Los Angeles, CA USA

**Keywords:** Alcoholic liver disease, Macrosteatosis, Sex dimorphism, Estrogen receptor, Preconditioning, Ischemia-reperfusion injury, Liver regeneration, Diseases, Gastroenterology, Health care, Medical research, Risk factors

## Abstract

A subset of deceased liver donors have a history of heavy alcohol intake. We evaluated whether donor heavy alcohol history is associated with graft failure after liver transplantation (LT) and whether associations differ by donor sex and macrosteatosis. We conducted a retrospective cohort study using the OPTN/UNOS registry, including 29,170 adult LT recipients (2000–2023). The exposure was heavy alcohol intake (> 2 drinks/day). The primary outcome was graft failure. Associations were estimated using Cox proportional hazards models adjusted for donor, recipient, and transplant covariates. The proportion of donors with heavy alcohol history increased over time (2000–2007, 19.0%; 2008–2015, 20.3%; 2016–2023, 22.3%). In multivariable analyses, donor heavy alcohol history was not associated with graft failure overall (HR = 0.96 [0.90–1.03], *P* = 0.249). Donor macrosteatosis was independently associated with higher risk (HR = 1.07 [1.01–1.13], *P* = 0.020). Associations differed by donor sex: among female donors, heavy alcohol history was associated with lower graft failure risk (HR = 0.87 [0.77–0.98], *P* = 0.010), whereas no association was observed among male donors (*P* = 0.802). The lower risk seen with female donors was not present when donor livers had macrosteatosis (*P* = 0.271). In conclusion, donor heavy alcohol history was not associated with increased graft failure risk overall. Notably, grafts from female donors with a heavy alcohol history were associated with lower failure risk, an apparent advantage that was not observed in the presence of macrosteatosis. No association was observed among male donors. These findings suggest sex- and liver-quality–dependent heterogeneity in the relationship between donor alcohol history and post-transplant outcomes.

**Supplementary Information:**

The online version contains supplementary material available at 10.1038/s41598-026-48596-y.

## Introduction

Alcohol intake can injure the liver through multiple mechanisms. In the Organ Procurement and Transplantation Network/United Network for Organ Sharing (OPTN/UNOS) registry, donor alcohol history is recorded based on surrogate interviews, and a standard drink is defined as 2 drinks (28 g of pure alcohol) or less in a day in accordance with National Institute on Alcohol Abuse and Alcoholism (NIAAA) guidance.^1–3^ In transplantation practice, living donors are advised to abstain from alcohol before liver donation to prioritize donor safety and optimize graft quality.^4^ By contrast, abstinence cannot be ensured for deceased donors; consequently, deceased donors with a history of heavy alcohol intake are not uncommon. Although many centers assume that donor alcohol history does not independently confer risk in deceased-donor liver transplantation (LT) once parenchymal quality meets accepted criteria^1,2^, practices vary in whether—and how much—this history is factored into donor selection.^5^.

Prior studies have largely reported overall associations between donor alcohol history and post-transplant outcomes.^1,2,5^ However, the hepatic effects of alcohol are complex and modified by individual factors.^6,7^ Women, in particular, are more vulnerable to alcohol-induced liver injury at comparable levels of intake.^8,9^ We therefore assessed whether the association between donor heavy alcohol history and graft failure differs by donor sex.

Alcohol exposure is a well-established risk factor for hepatic macrosteatosis, a clinically consequential parenchymal change that influences graft selection and outcomes.^6,10^ We hypothesized that any association between donor alcohol history and graft failure would be modified by the presence of donor macrosteatosis. To address these questions, we analyzed contemporary OPTN/UNOS data.

## Methods

### Study design and data source

We conducted a retrospective cohort study using data from the OPTN, supplied by the UNOS. Data files were created based on the dataset as of October 6, 2023. Donor alcohol history was obtained from family/friends during the donor risk assessment interview. Interpretation and reporting are solely the authors’ responsibility and do not represent official policy or interpretation by OPTN, UNOS, or the U.S. Government. The dataset used and/or analyzed during the current study is available from the corresponding author upon reasonable request. The Institutional Review Board of Samsung Medical Center approved the study (SMC 2024-10-012) and waived written informed consent. The study adhered to the Declaration of Helsinki and Good Clinical Practice guidelines.

### Study population

**Fig. 1:**
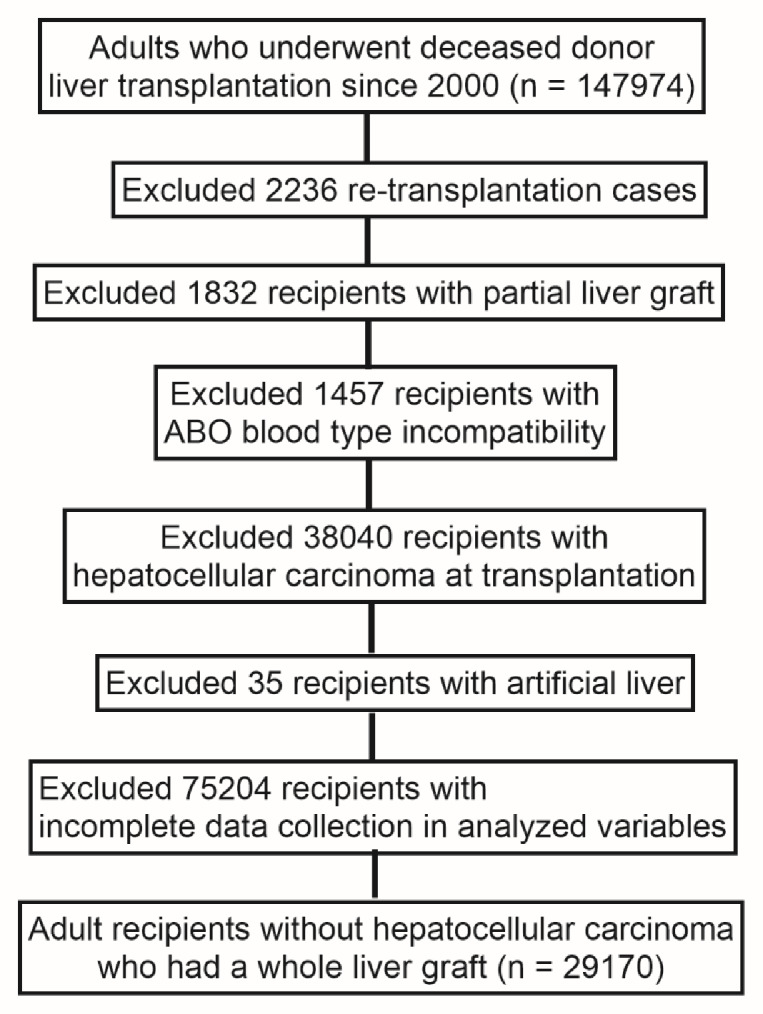
Study cohort selection (OPTN/UNOS 2000–2023). Flow diagram showing inclusion/exclusion of adult deceased-donor liver transplant recipients and the final analytic cohort (*n* = 29,170). Counts at each step are displayed.

Among 357,269 patients registered on the UNOS waiting list from 1987 to 2023, we identified 147,974 adults who underwent deceased-donor LT from 2000 onward (Fig. 1). We excluded 2,236 re-transplantations, 1,832 partial (split) grafts, and 1,457 ABO-incompatible transplants, leaving 142,449 recipients. We then excluded 38,040 recipients with hepatocellular carcinoma (HCC) at transplantation considering the differences in the effects of donor sex and macrosteatosis on HCC-related graft failure and HCC-unrelated graft failure^11–13^, yielding 104,409 recipients. After excluding 35 recipients with artificial liver support, 18,519 with missing donor alcohol history, 56,048 with missing graft macrosteatosis data, and 295 with missing graft survival data, 29,512 recipients remained. Finally, 342 recipients were excluded due to missing covariates—primary liver disease (*n* = 8), diabetes (*n* = 200), prior upper abdominal surgery (*n* = 13), life-support device use (*n* = 2), dialysis (*n* = 113), and encephalopathy grade (*n* = 6)—resulting in a final cohort of 29,170 non-HCC adults who received whole-liver grafts from deceased donors.

### Exposure and covariates

The exposure of interest was donor heavy alcohol intake, defined in the UNOS/OPTN data as > 2 drinks/day.^2^ For the purposes of this study, macrosteatosis was defined as > 5% of hepatocytes containing macrovesicular fat droplets, consistent with nonalcoholic fatty liver disease activity score conventions^14,15^ and prior reports indicating differences in hepatic estrogen receptor content at the 5% threshold^10,16^. Donor, recipient, and transplant characteristics used as covariates are listed in the main tables; complete-case analysis was performed.

### Statistical analysis

The primary outcome was graft failure, defined as death or re-transplantation, whichever occurred first. Follow-up was administratively censored at 5 years post-transplant or at last known follow-up, whichever came first. Time-to-event associations were estimated using Cox proportional hazards models, and results are reported as hazard ratios (HRs) with 95% confidence intervals. We tested effect modification by including an interaction term between heavy alcohol use and donor sex in the Cox model. Pre-specified subgroup analyses evaluated whether associations differed by donor sex and by donor macrosteatosis status. All variables examined in univariable analyses were entered into multivariable models. Continuous variables are summarized as median (interquartile range) and categorical variables as number (percentage). All tests were two-sided, with *P* < 0.05 considered statistically significant. Analyses were performed using IBM SPSS Statistics, version 29.0 (IBM Corp., Armonk, NY, USA).

## Results

### Donor alcohol history and baseline characteristics

Heavy alcohol intake was more frequent in male donors than in female donors (27.6% vs. 13.5%; *P* < 0.001). The proportion of donors with heavy alcohol history increased across eras: 2000–2007, 19.0%; 2008–2015, 20.3%; and 2016–2023, 22.3%. This pattern was observed in both sexes (female: 11.4%, 12.7%, 14.5%; male: 25.8%, 27.1%, 28.2%). Macrosteatosis was more frequent among donors with heavy alcohol intake than among those without, both in female donors (62.8% vs. 59.9%; *P* = 0.024) and in male donors (65.3% vs. 60.3%; *P* < 0.001). Baseline characteristics according to donor alcohol history in the overall cohort are summarized in Table 1.

**Table 1:** Baseline clinical characteristics of recipients according to donor alcohol history.

	Non-heavy intake (*n* = 22983)	Heavy intake (*n* = 6187)	*P* value
Donor age (y)	49 (36–59)	49 (40–57)	0.028
Donor sex (female)	11,406 (49.6)	1780 (28.8)	< 0.001
Donor body mass index (kg/m^2^)	29.1 (24.8–34.5)	26.7 (23.5–30.9)	< 0.001
Donor cause of death			< 0.001
Anoxia	8581 (37.3)	2269 (36.7)	
Cerebrovascular/stroke	9466 (41.2)	2114 (34.2)	
Head trauma/others	4936 (21.5)	1804 (29.2)	
Graft macrosteatosis			< 0.001
≤ 5%	14,816 (64.5)	3664 (59.2)	
5%-30%	7214 (31.4)	2210 (35.7)	
> 30%	953 (4.1)	313 (5.1)	
Cold ischemia time (h)	6.2 (5.0–8.0)	6.2 (5.0-7.9)	0.444
Transplant era			< 0.001
2000–2007	3091 (13.4)	724 (11.7)	
2008–2015	7343 (31.9)	1871 (30.2)	
2016–2023	12,549 (54.6)	3592 (58.1)	
Recipient age (y)	55 (48–62)	55 (47–61)	0.559
Recipient sex (female)	14,549 (63.3)	3966 (64.1)	0.247
Body mass index (kg/m^2^)	28.0 (24.3–32.4)	28.2 (24.6–32.6)	0.304
Diabetes	5963 (25.9)	1600 (25.9)	0.893
Primary liver disease			0.043
Viral	4077 (17.7)	1138 (18.4)	
Alcoholic	7804 (34.0)	2183 (35.3)	
MASH	3928 (17.1)	994 (16.1)	
Others	7174 (31.2)	1872 (30.3)	
Hepatic encephalopathy III-IV	3352 (14.6)	890 (14.4)	0.692
Refractory ascites	8853 (38.5)	2419 (39.1)	0.407
Previous upper abdominal surgery	10,676 (46.5)	2802 (45.3)	0.103
Portal vein thrombosis	2673 (11.6)	696 (11.2)	0.405
MELD score	24 (18–31)	24 (18–31)	0.412
Pretransplant critical care			
Dialysis	3460 (15.1)	956 (15.5)	0.439
Life-supporting device	1839 (8.0)	494 (8.0)	0.965
Mechanical ventilation	1015 (4.4)	238 (3.8)	0.050
Pretransplant biochemical status			
Albumin (g/dL)	3.0 (2.5–3.5)	3.0 (2.6–3.5)	0.745
Total bilirubin (mg/dL)	5.0 (2.4–12.2)	5.0 (2.4–12.1)	0.260
Creatinine (mg/dL)	1.20 (0.85–1.90)	1.20 (0.82–1.90)	0.450
Prothrombin time (INR)	1.80 (1.40–2.38)	1.80 (1.40–2.40)	0.415

Data are presented as median (interquartile range) or frequency (%). INR, international normalized ratio; MASH, metabolic dysfunction-associated steatohepatitis; MELD, model for end-stage liver disease.

### Overall association with graft failure

**Fig. 2:**
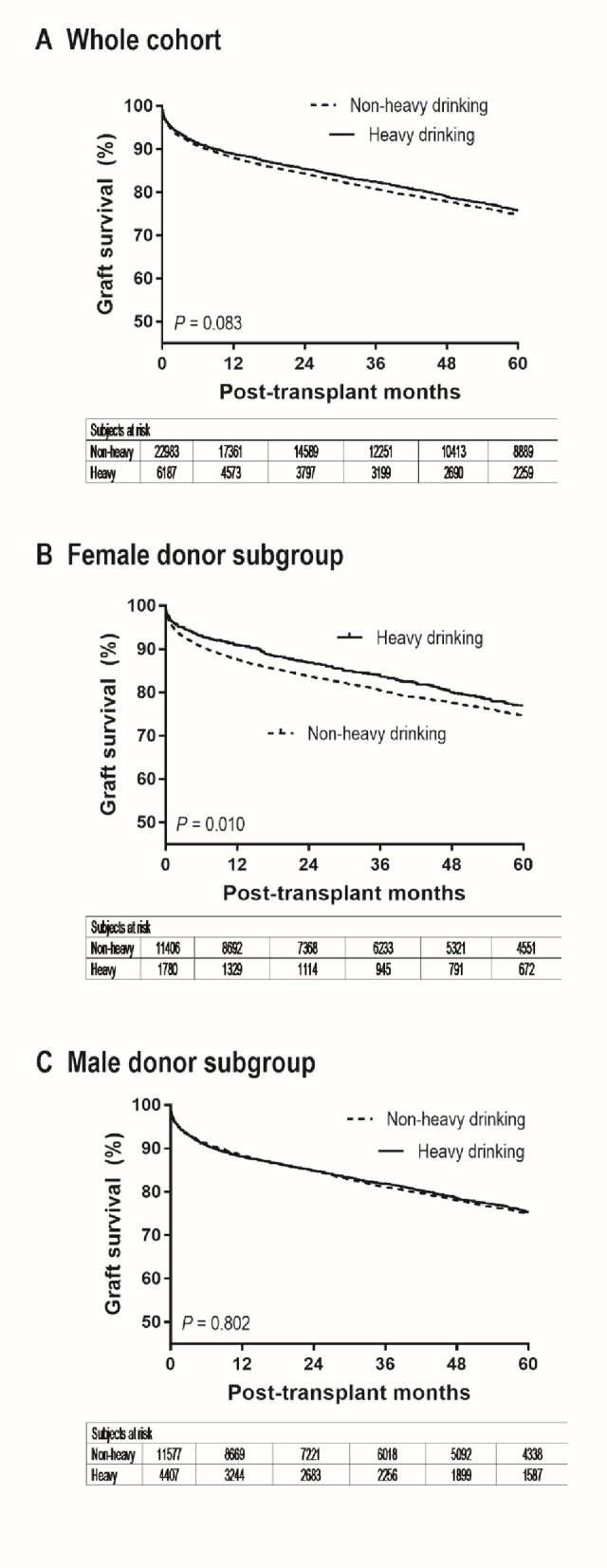
Graft survival by donor heavy alcohol history in (**A**) overall cohort, (**B**) female-donor subgroup, and (**C**) male-donor subgroup. P values are from univariable tests. (Adjusted associations are reported in Table 2.)

The results of univariable analysis demonstrated no significant difference in graft failure between donors with vs. without heavy alcohol history (Fig. 2A and Supplementary Table S1; HR = 0.95 [0.89–1.01], *P* = 0.083). In multivariable analysis, donor heavy alcohol history was not associated with graft failure (HR = 0.96 [0.90–1.03], *P* = 0.249), whereas donor macrosteatosis was independently associated with higher risk (HR = 1.07 [1.01–1.13], *P* = 0.020) (Table 2). There was a significant interaction between donor heavy alcohol use and donor sex in univariable analysis (*P* = 0.047) and multivariable analysis (*P* = 0.037), indicating that the association between donor heavy alcohol use and graft failure differed by donor sex. The interaction term corresponded to a HR of 0.87 (0.75–0.99) and 0.86 (0.74–0.99), respectively.

**Table 2:** Multivariable analysis for graft failure in the overall cohort.

	Hazard ratio	*P* value
Donor heavy alcohol intake	0.95 (0.89–1.02)	0.142
Donor age (y)	1.01 (1.01–1.01)	< 0.001
Donor sex (male vs. female)	1.01 (0.96–1.07)	0.699
Donor body mass index (kg/m^2^)	1.00 (0.99-1.00)	0.009
Donor cause of death (vs. Anoxia)		
Cerebrovascular/stroke	1.13 (1.05–1.20)	< 0.001
Head trauma/others	1.04 (0.96–1.12)	0.329
Graft macrosteatosis > 5%	1.08 (1.02–1.14)	0.008
Cold ischemia time (h)	1.03 (1.02–1.04)	< 0.001
Transplant era (vs. 2000–2007)		
2008–2015	0.68 (0.63–0.73)	< 0.001
2016–2023	0.57 (0.53–0.62)	< 0.001
Recipient age (y)	1.01 (1.00-1.01)	< 0.001
Recipient sex (male vs. female)	1.13 (1.07–1.20)	< 0.001
Body mass index (kg/m^2^)	1.00 (1.00-1.01)	0.642
Diabetes	1.26 (1.19–1.34)	< 0.001
Primary liver disease (vs. viral)		
Alcoholic	0.82 (0.76–0.89)	< 0.001
MASH	0.80 (0.73–0.88)	< 0.001
Others	0.92 (0.86–0.99)	0.032
Hepatic encephalopathy III-IV	1.22 (1.13–1.32)	< 0.001
Refractory ascites	1.01 (0.95–1.07)	0.853
Previous upper abdominal surgery	1.25 (1.18–1.32)	< 0.001
Portal vein thrombosis	1.24 (1.15–1.34)	< 0.001
MELD score	1.01 (1.00-1.01)	0.067
Dialysis	1.09 (0.99–1.19)	0.077
Life-supporting device	1.49 (1.35–1.64)	< 0.001
Mechanical ventilation	1.13 (0.97–1.31)	0.121
Albumin (g/dL)	0.93 (0.90–0.97)	< 0.001
Total bilirubin (mg/dL)	1.00 (1.00-1.01)	0.156
Creatinine (mg/dL)	1.04 (1.02–1.06)	< 0.001
Prothrombin time (INR)	0.99 (0.96–1.02)	0.454

INR, international normalized ratio; MASH, model for end-stage liver disease; MELD, model for end-stage liver disease.

### Female donor subgroup

Baseline characteristics stratified by donor sex (female vs. male) and donor alcohol history (non-heavy vs. heavy intake) are summarized in Table 3. Among recipients of grafts from female donors, heavy alcohol history was associated with a significantly lower risk of graft failure (Fig. 2B; HR = 0.86 [0.76–0.97], *P* = 0.011). Graft survival probabilities at 1, 2, and 5 years were 87.7% (95% confidence interval; 87.0%–88.3%), 83.8% (83.0%–84.5%), and 74.7% (73.8%–75.6%) without donor heavy alcohol history, versus 90.9% (89.4%–92.2%), 86.8% (85.0%–88.4%), and 76.9% (74.5%–79.1%) with donor heavy alcohol history.

**Table 3:** Baseline clinical characteristics of recipients stratified by donor sex and donor alcohol history.

	Female donor			Male donor		
	Non-heavy intake (*n* = 11406)	Heavy intake (*n* = 1780)	*P* value	Non-heavy intake (*n* = 11577)	Heavy intake (*n* = 4407)	*P* value
Donor age (y)	50 (39–60)	49 (41–57)	0.160	47 (34–59)	49 (39–57)	< 0.001
Donor body mass index (kg/m^2^)	29.8 (24.9–36.1)	26.6 (23.1–31.5)	< 0.001	28.6 (24.7–33.2)	26.7 (23.7–30.8)	< 0.001
Donor cause of death			< 0.001			< 0.001
Anoxia	4364 (38.3)	739 (41.5)		4217 (36.4)	1530 (34.7)	
Cerebrovascular/stroke	5438 (47.7)	724 (40.7)		4028 (34.8)	1390 (31.5)	
Head trauma/others	1604 (14.1)	317 (17.8)		3332 (28.8)	1487 (33.7)	
Macrosteatosis > 5%	6837 (59.9)	1117 (62.8)	0.024	6981 (60.3)	2877 (65.3)	< 0.001
Cold ischemia time (h)	6.2 (5.0–8.0)	6.3 (5.1–7.9)	0.052	6.3 (5.0–8.0)	6.2 (5.0-7.9)	< 0.007
Transplant era			< 0.001			0.068
2000–2007	1601 (14.0)	205 (11.5)		1490 (12.9)	519 (11.8)	
2008–2015	3796 (33.3)	552 (31.0)		3547 (30.6)	1319 (29.9)	
2016–2023	6009 (52.7)	1023 (57.5)		6540 (56.5)	2569 (58.3)	
Recipient age (y)	55 (48–62)	55 (47–61)	0.266	55 (48–61)	55 (48–61)	0.816
Recipient sex (female)	4907 (43.0)	797 (44.8)	0.165	3527 (30.5)	1424 (32.3)	0.024
Body mass index (kg/m^2^)	27.7 (24.1–32.2)	27.8 (24.0-32.3)	0.568	28.4 (24.6–32.7)	28.3 (24.8–32.8)	0.940
Diabetes	2948 (25.8)	455 (25.6)	0.799	3015 (36.0)	1145 (26.0)	0.937
Primary liver disease			0.667			0.350
Viral	2021 (17.7)	334 (18.8)		2056 (17.8)	804 (18.2)	
Alcoholic	3656 (32.1)	576 (32.4)		4148 (35.8)	1607 (36.5)	
MASH	1962 (17.2)	296 (16.6)		1966 (17.0)	698 (15.8)	
Others	3767 (33.0)	574 (32.2)		3407 (29.4)	1298 (29.5)	
Hepatic encephalopathy III-IV	1665 (14.6)	264 (14.8)	0.795	5401 (39.1)	1579 (39.5)	0.555
Refractory ascites	4333 (38.0)	686 (38.5)	0.656	4520 (39.0)	1733 (39.3)	0.745
Previous upper abdominal surgery	5497 (48.2)	859 (48.3)	0.960	5179 (44.7)	1943 (44.1)	0.463
Portal vein thrombosis	1274 (11.2)	200 (11.2)	0.934	1399 (12.1)	496 (11.3)	0.147
MELD score	24 (18–31)	24 (18–32)	0.977	24 (18–31)	24 (18–31)	0.711
Pretransplant critical care						
Dialysis	1690 (14.8)	273 (15.3)	0.566	1770 (15.3)	683 (15.5)	0.743
Life-supporting device	884 (7.8)	134 (7.5)	0.744	955 (8.2)	360 (8.2)	0.869
Mechanical ventilation	509 (4.5)	61 (3.4)	0.046	506 (4.4)	177 (4.0)	0.322
Pretransplant biochemical status						
Albumin (g/dL)	3.0 (2.5–3.5)	3.0 (2.6–3.5)	0.245	3.0 (2.6–3.5)	3.0 (2.5–3.5)	0.729
Total bilirubin (mg/dL)	5.1 (2.5–12.5)	5.1 (2.3–13.1)	0.669	4.8 (2.4–11.9)	4.9 (2.4–11.8)	0.555
Creatinine (mg/dL)	1.20 (0.83–1.86)	1.18 (0.80–1.92)	0.607	1.20 (0.86–1.90)	1.20 (0.83–1.90)	0.944
Prothrombin time (INR)	1.80 (1.40–2.39)	1.80 (1.40–2.39)	0.779	1.80 (1.40–2.37)	1.80 (1.40–2.40)	0.946

Data are presented as median (interquartile range) or frequency (%).INR, international normalized ratio; MASH, metabolic dysfunction-associated steatohepatitis; MELD, model for end-stage liver disease.

### Male donor subgroup

Among recipients of grafts from male donors, graft failure risk did not differ by donor alcohol history (Fig. 2C; HR = 0.99 [0.92–1.07], *P* = 0.802). Graft survival probabilities at 1, 2, and 5 years were 88.5% (87.8%–89.0%), 84.9% (84.1%–85.5%), and 75.0% (74.1%–76.0%) without donor heavy alcohol history, and 88.2% (87.1%–89.1%), 84.9% (83.7%–86.0%), and 75.3% (73.7%–76.7%) with donor heavy alcohol history.

### Interaction with macrosteatosis among female donors

**Fig. 3:**
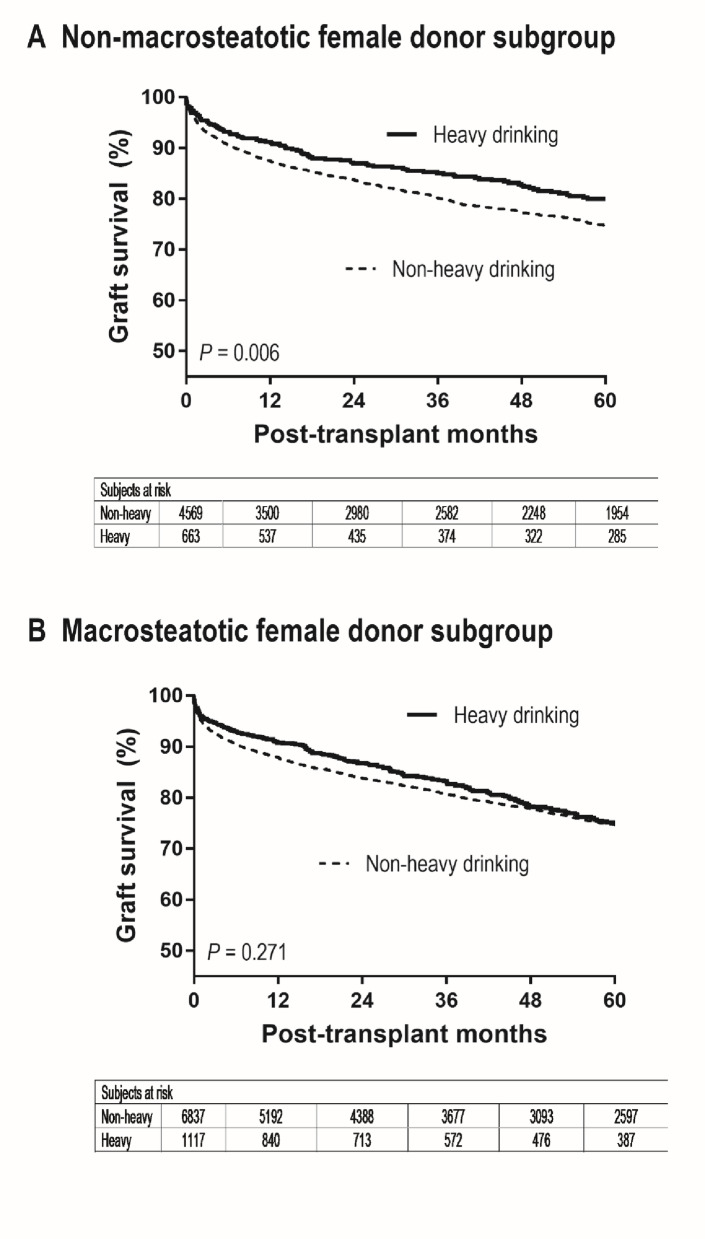
Graft survival by donor heavy alcohol history among female-donor grafts, stratified by macrosteatosis: (**A**) non-macrosteatotic grafts (≤ 5% macrovesicular steatosis) and (**B**) macrosteatotic grafts (> 5%). P values are from univariable tests(Adjusted associations are reported in Table 4.)

In the female donor subgroup, the interaction between heavy alcohol use and macrosteatosis was significant (*P* = 0.048) and the interaction term corresponded to a HR of 0.78 (0.60–0.99) in the multivariable Cox model, indicating that the association between donor heavy alcohol use and graft failure differed by donor macrosteatosis. Among female donors with macrosteatosis, graft failure risk did not differ by alcohol history (HR = 0.92 [0.80–1.07], *P* = 0.271) (Fig. 3). Among female donors without macrosteatosis, heavy alcohol history was associated with lower graft failure risk (Supplementary Table S2; HR = 0.76 [0.62–0.92], *P* = 0.006). Corresponding 1-, 2-, and 5-year graft survival probabilities were 87.4% (86.4%–88.4%), 83.7% (82.5%–84.8%), and 74.8% (73.3%–76.1%) without donor heavy alcohol history, versus 91.2% (88.7%–93.2%), 87.0% (84.0%–89.4%), and 80.0% (76.2%–83.2%) with donor heavy alcohol history. Multivariable analysis confirmed the association in non-macrosteatotic female donor grafts (HR = 0.72 [0.58–0.88], *P* = 0.002) (Table 4).

**Table 4:** Multivariable analysis for graft failure in the subgroup of recipients with non-macrosteatotic female donor.

	Hazard ratio	*P* value
Donor heavy alcohol intake	0.72 (0.58–0.88)	0.002
Donor age (y)	1.00 (1.00-1.01)	0.049
Donor body mass index (kg/m^2^)	0.99 (0.99-1.00)	0.079
Donor cause of death (vs. Anoxia)		
Cerebrovascular/stroke	1.14 (0.98–1.32)	0.087
Head trauma/others	0.89 (0.74–1.09)	0.267
Cold ischemia time (h)	1.03 (1.01–1.05)	0.012
Transplant era (vs. 2000–2007)		
2008–2015	0.71 (0.60–0.84)	< 0.001
2016–2023	0.62 (0.52–0.75)	< 0.001
Recipient age (y)	1.01 (1.00-1.01)	0.057
Recipient sex (male vs. female)	1.15 (1.01–1.31)	0.032
Body mass index (kg/m^2^)	0.99 (0.98-1.00)	0.239
Diabetes	1.33 (1.15–1.53)	< 0.001
Primary liver disease (vs. viral)		
Alcoholic	0.93 (0.78–1.12)	0.446
MASH	0.86 (0.69–1.07)	0.180
Others	0.98 (0.85–1.16)	0.770
Hepatic encephalopathy III-IV	1.20 (1.01–1.43)	0.042
Refractory ascites	1.00 (0.87–1.14)	0.962
Previous upper abdominal surgery	1.22 (1.07–1.38)	0.002
Portal vein thrombosis	1.32 (1.10–1.58)	0.003
MELD score	1.00 (0.99–1.02)	0.866
Dialysis	1.32 (1.07–1.62)	0.009
Life-supporting device	1.65 (1.32–2.06)	< 0.001
Mechanical ventilation	1.01 (0.72–1.42)	0.960
Albumin (g/dL)	0.90 (0.83–0.99)	0.024
Total bilirubin (mg/dL)	1.00 (0.99–1.01)	0.958
Creatinine (mg/dL)	1.03 (0.98–1.08)	0.293
Prothrombin time (INR)	1.02 (0.97–1.07)	0.512

INR, international normalized ratio; MASH, model for end-stage liver disease; MELD, model for end-stage liver disease.

## Discussion

In this national cohort study of 29,170 adult deceased-donor LT recipients, donor heavy alcohol history was not associated with increased risk of graft failure overall. Two context-specific findings emerged. First, grafts from female donors with a heavy alcohol history were associated with lower graft failure risk, whereas no association was observed among male donors. This finding is important because female alcohol intake has been increasing and the use of female donors with heavy alcohol intake can raise concerns due to the vulnerability of women to alcohol toxicity.^17,18^ Second, the lower risk among female donors was absent when donor livers had macrosteatosis. This finding is important because heavy alcohol intake is associated with the development of hepatic steatosis.^19^ These results are timely, given that the proportion of deceased donors with a heavy alcohol history increased over the study period and that centers vary in how this information is used during donor selection. In agreement with prior literature^1,2,5^, our overall null finding supports the common practice of prioritizing pathologic parenchymal quality over alcohol history per se. Importantly, we extend prior work by showing sex-specific heterogeneity and modification by liver quality.

The liver is the main organ responsible for metabolizing alcohol; accordingly, is also the primary target injured by alcohol and its metabolites.^8,9^ For example, as an end product of the oxidative pathway metabolizing ethanol, acetaldehyde damages the liver by directly triggering necrosis, inflammation, extracellular matrix remodeling, and fibrogenesis. Clinically, it is well known that chronic alcohol intake leads to the infiltration of fat in the liver, the so-called steatosis, a mild and reversible form of alcohol-induced liver disease.^8,9^ Liver steatosis is associated with the decrease in the tolerance to ischemia-reperfusion injury and regenerative capacity.^20,21^ Thus, we had thought at the beginning of the study that donor heavy alcohol intake may negatively affect graft survival particularly with specific conditions (e.g. female sex, macrosteatosis). However, graft failure risk decreased in relation to donor alcohol intake with female donors. The favorable effect of donor alcohol intake might be attributable to the preconditioning effect of alcohol.^22,23^ That is, like other detrimental hepatic insults including toxins and drugs^24^, alcohol induces various protective mechanisms to improve liver regeneration capacity and recovery from the injuries. The protection mechanisms can act as a preconditioning before a more profound injury (i.e. hepatectomy or liver transplantation) and help the liver endure the hepatic insults.^22,25,26^ In terms of the sex difference, a plausible explanation can be found in the sex disparity in the action of alcohol. There have been evidence that some degree of alcohol intake does not influence male livers, whereas the same amount influences female livers.^27,28^ Women develop progressive alcoholic liver disease more readily, and at a lower dose, than do males.^29^ It has been reported that a daily consumption of even 20 g in women leads to an advanced form of liver disease while 60–80 g is required in men. Because heavy alcohol intake defined in the UNOS/OPTN data is equivalent to > 28 g/d of pure alcohol , it can be deduced that this amount is not enough to generate hepatic reaction in the liver from a male donor while the liver from a female donor reacts to it^30^.

The change of the benefit of donor heavy alcohol intake by macrosteatosis suggested that the dominant effect of macrosteatosis overrides alcohol intake and macrosteatotic liver requires another type or degree of insult to generate preconditioning mechanisms.^26,31,32^ The content of estrogen receptor is known to decrease in macrosteatotic livers than in normal livers in women.^6,10^ Because the liver is extremely sensitive to the action of estrogen and hepatic estrogen signaling plays an important role in mitigating injury and enhancing recovery of the liver^33,34,35^, it could be deduced that the change in hepatic estrogen signaling in macrosteatotic donor liver is associated with the change of the preconditioning effects of heavy alcohol intake. The interaction with macrosteatosis also suggested that donor alcohol intake does not impose additional risk on macrosteatosis, suggesting that graft failure risk does not differ by the origin of graft macrosteatosis (alcohol-induced macrosteatosis vs. metabolic dysfunction-associated macrosteatosis).

This study has several limitations. First, this retrospective registry analysis is subject to residual confounding and selection bias. In particular, extensive exclusion due to missing donor alcohol history and donor macrosteatosis data raises concerns regarding selection bias and generalizability. For instance, as shown in Supplementary Table S3, there were significant differences between patients excluded due to missing macrosteatosis data and those included in the analysis, suggesting that macrosteatosis assessment may be influenced by donor quality, center practice, and procurement logistics. Although covariates were adjusted for in multivariable analyses, registry variables may be insufficient to account for many clinically relevant donor and procurement factors. Second, donor alcohol history was obtained from surrogate interviews and may be misclassified; the database also lacks information on duration, patterns (e.g., binge vs. daily), and lifetime exposure. The OPTN/UNOS categories do not distinguish never-drinkers from mild drinkers. Nonetheless, our data is still important because NIAAA guideline indicates that the impact of alcohol intake is not based on an average amount over several days but rather the amount consumed on any single day.^3^ Third, the mechanisms underlying the interaction between donor alcohol intake, sex, and macrosteatosis is beyond the scope of the current study and the suggested mechanisms are speculative and not directly supported by the presented data without biomarkers, histologic severity gradients, ischemia-reperfusion metrics, and regeneration markers as well as systemic hormone data and hepatic estrogen receptor expression. Fourth, these results may not be applicable to living donation, for which pre-donation abstinence is recommended to ensure donor safety. This study suggests that using grafts from deceased donors with heavy alcohol use, for whom a period of pre-donation abstinence is not feasible, may be safe in the context of organ shortage; however, these findings should not be interpreted as endorsing heavy alcohol consumption. Fifth, the exact definition of “life support” and “artificial liver” is unclear. Life support at transplant was defined using the OPTN/UNOS variable LIFE_SUP_TRR (Patient on life support: yes/no). When life support was present, the TRR form records the modality, including artificial liver (captured as ARTIFIC_LIVER_TRR), mechanical ventilation, and other mechanisms specified by the transplant center. The OPTN/UNOS TRR data collection materials list “Artificial Liver” as a life-support modality but do not further specify the exact device or extracorporeal liver support technique encompassed by this item.

In conclusion, donor heavy alcohol history was not associated with increased graft failure risk overall. Among female donors, heavy alcohol history was associated with lower risk—an association that was not observed in the presence of macrosteatosis—and no association was seen among male donors. These findings underscore sex- and liver-quality–dependent heterogeneity in the relationship between donor alcohol history and post-transplant outcomes and support donor selection practices that prioritize parenchymal quality.

## Supplementary Information

Below is the link to the electronic supplementary material.

Supplementary Material 1

## Data Availability

The data have been supplied by the UNOS as the contractor for the OPTN. The database used and/or analyzed during the current study is available from the corresponding author on reasonable request.

## Abbreviations

HCC: Hepatocellular carcinoma
HR: Hazard ratio
LT: Liver transplantation
NIAAA: National Institute on Alcohol Abuse and Alcoholism
OPTN: Organ Procurement and Transplantation Network
UNOS: United Network for Organ Sharing
