# Supplementary material for: Association of donor heavy alcohol use with graft failure after deceased-donor liver transplantation stratified by donor sex and macrosteatosis in the OPTN/UNOS registry
Source: Sci Rep. 2026 Apr 15;16:17502. doi: 10.1038/s41598-026-48596-y (PMC13237369; doi:10.1038/s41598-026-48596-y)
Supplement: Supplementary file 1 — Supplementary Material 1 [file 41598_2026_48596_MOESM1_ESM.docx]

**Supplementary Table S1.** Univariable analysis for the effect on graft failure risk

|  | **Hazard ratio** | **P value** |
| --- | --- | --- |
| Donor heavy alcohol intake | 0.95 (0.89-1.01) | 0.083 |
| Donor age (y) | 1.01 (1.01-1.01) | <0.001 |
| Donor sex (male vs. female) | 0.99 (0.94-1.04) | 0.651 |
| Donor body mass index (kg/m^2^) | 0.99 (0.99-1.00) | <0.001 |
| Donor cause of death (vs. Anoxia) |  |  |
| Cerebrovascular/stroke | 1.36 (1.28-1.45) | <0.001 |
| Head trauma/others | 1.15 (1.07-1.23) | <0.001 |
| Graft macrosteatosis > 5% | 1.05 (1.00-1.11) | 0.056 |
| Cold ischemia time (h) | 1.04 (1.03-1.05) | <0.001 |
| Transplant era (vs. 2000-2007) |  |  |
| 2008-2015 | 0.68 (0.63-0.72) | <0.001 |
| 2016-2023 | 0.52 (0.48-0.55) | <0.001 |
| Recipient age (y) | 1.01 (1.00-1.01) | <0.001 |
| Recipient sex (male vs. female) | 1.07 (1.02-1.13) | 0.010 |
| Body mass index (kg/m^2^) | 1.00 (1.00-1.01) | 0.348 |
| Diabetes | 1.29 (1.22-1.36) | <0.001 |
| Primary liver disease (vs. viral) |  |  |
| Alcoholic | 0.72 (0.67-0.78) | <0.001 |
| MASH | 0.77 (0.71-0.84) | <0.001 |
| Others | 0.88 (0.82-0.94) | <0.001 |
| Hepatic encephalopathy III-IV | 1.36 (1.27-1.46) | <0.001 |
| Refractory ascites | 1.07 (1.01-1.12) | 0.016 |
| Previous upper abdominal surgery | 1.20 (1.14-1.26) | <0.001 |
| Portal vein thrombosis | 1.20 (1.11-1.29) | <0.001 |
| MELD score | 1.01 (1.01-1.01) | <0.001 |
| Dialysis | 1.32 (1.23-1.41) | <0.001 |
| Life-supporting device | 1.65 (1.53-1.79) | <0.001 |
| Mechanical ventilation | 1.67 (1.47-1.90) | <0.001 |
| Albumin (g/dL) | 0.91 (0.87-0.94) | <0.001 |
| Total bilirubin (mg/dL) | 1.01 (1.00-1.01) | <0.001 |
| Creatinine (mg/dL) | 1.07 (1.06-1.09) | <0.001 |
| Prothrombin time (INR) | 1.01 (0.99-1.03) | 0.231 |

INR, international normalized ratio; MASH, model for end-stage liver disease; MELD, model for end-stage liver disease.

**Supplementary Table S2.** Univariable analysis in the subgroup of recipients with non-macrosteatotic female donor

|  | **Hazard ratio** | **P value** |
| --- | --- | --- |
| Donor heavy alcohol intake | 0.76 (0.62-0.92) | 0.006 |
| Donor age (y) | 1.01 (1.00-1.01) | 0.003 |
| Donor body mass index (kg/m^2^) | 0.99 (0.98-1.00) | 0.046 |
| Donor cause of death (vs. Anoxia) |  |  |
| Cerebrovascular/stroke | 1.28 (1.12-1.47) | <0.001 |
| Head trauma/others | 0.97 (0.80-1.17) | 0.750 |
| Cold ischemia time (h) | 1.04 (1.02-1.06) | <0.001 |
| Transplant era (vs. 2000-2007) |  |  |
| 2008-2015 | 0.71 (0.61-0.83) | <0.001 |
| 2016-2023 | 0.59 (0.51-0.70) | <0.001 |
| Age (y) | 1.01 (1.00-1.01) | 0.061 |
| Recipient sex (male vs. female) | 1.09 (0.97-1.23) | 0.151 |
| Body mass index (kg/m^2^) | 1.00 (0.99-1.01) | 0.381 |
| Diabetes | 1.33 (1.17-1.52) | <0.001 |
| Primary liver disease (vs. viral) |  |  |
| Alcoholic | 0.82 (0.69-0.97) | 0.020 |
| MASH | 0.82 (0.67-1.00) | 0.050 |
| Others | 0.93 (0.79-1.09) | 0.353 |
| Hepatic encephalopathy III-IV | 1.41 (1.21-1.65) | <0.001 |
| Refractory ascites | 1.06 (0.94-1.20) | 0.351 |
| Previous upper abdominal surgery | 1.18 (1.04-1.33) | 0.008 |
| Portal vein thrombosis | 1.27 (1.07-1.52) | 0.007 |
| MELD score | 1.01 (1.00-1.02) | 0.004 |
| Dialysis | 1.50 (1.29-1.75) | <0.001 |
| Life-supporting device | 1.87 (1.57-2.23) | <0.001 |
| Mechanical ventilation | 1.71 (1.29-2.27) | <0.001 |
| Albumin (g/dL) | 0.90 (0.83-0.98) | 0.018 |
| Total bilirubin (mg/dL) | 1.00 (1.00-1.01) | 0.229 |
| Creatinine (mg/dL) | 1.07 (1.03-1.11) | <0.001 |
| Prothrombin time (INR) | 1.03 (0.99-1.07) | 0.187 |

INR, international normalized ratio; MASH, model for end-stage liver disease; MELD, model for end-stage liver disease.

**Supplementary Table S3.** Comparison of characteristics between patients with and without macrosteatosis degree data.

|  | **With macrosteatosis data (n=29807)** | **Without macrosteatosis data (n=56048)** | **P value** |
| --- | --- | --- | --- |
| Donor heavy alcohol intake | 6331 (21.2) | 7534 (13.4) | <0.001 |
| Donor age (y) | 49 (37-59) | 35 (24-49) | <0.001 |
| Donor sex (male) | 16337 (54.8) | 35301 (63.0) | <0.001 |
| Donor body mass index (kg/m^2^) | 28.5 (24.4-33.8) | 25.8 (22.7-29.4) | <0.001 |
| Donor cause of death |  |  | <0.001 |
| Anoxia | 11137 (37.4) | 16860 (30.1) |  |
| Cerebrovascular/stroke | 11815 (39.6) | 16144 (28.8) |  |
| Head trauma/others | 6855 (23.0) | 23044 (41.1) |  |
| Cold ischemia time (h) | 6.22 (5.00-8.00) | 6.00 (4.75-7.55) | <0.001 |
| Transplant era |  |  | <0.001 |
| 2000-2007 | 3907 (13.1) | 11173 (19.9) |  |
| 2008-2015 | 9326 (31.3) | 19260 (34.4) |  |
| 2016-2023 | 16574 (55.6) | 25615 (45.7) |  |
| Recipient age (y) | 55 (48-61) | 54 (45-60) | <0.001 |
| Recipient sex (male) | 18921 (63.5) | 33873 (60.4) | <0.001 |
| Body mass index (kg/m^2^) | 28.1 (24.4-32.5) | 27.5 (23.9-32.1) | <0.001 |
| Diabetes | 7659 (25.7) | 13284 (23.7) | <0.001 |
| Hepatic encephalopathy III-IV | 4343/29799 (14.6) | 9568/56017 (17.1) | <0.001 |
| Refractory ascites | 11507/29799 (38.6) | 21801/56017 (38.9) | 0.386 |
| Previous upper abdominal surgery | 13612/29531 (46.1) | 25344/55360 (45.8) | 0.382 |
| Portal vein thrombosis | 3423/29531 (11.6) | 5858/55366 (10.6) | <0.001 |
| MELD score | 24 (18-31) | 27 (19-34) | <0.001 |
| Dialysis | 4502/29689 (15.2) | 11904/55901 (21.3) | <0.001 |
| Life-supporting device | 2376/29542 (8.0) | 6699/55385 (12.1) | <0.001 |
| Mechanical ventilation | 772 (2.6) | 2506 (4.5) | <0.001 |
| Albumin (g/dL) | 3.00 (2.60-3.50) | 3.00 (2.50-3.50) | 0.264 |
| Total bilirubin (mg/dL) | 5.00 (2.40-12.20) | 6.40 (2.70-17.26) | <0.001 |
| Creatinine (mg/dL) | 1.20 (0.84-1.90) | 1.30 (0.88-2.20) | <0.001 |
| Prothrombin time (INR) | 1.80 (1.40-2.38) | 1.89 (1.47-2.50) | <0.001 |

INR, international normalized ratio; MASH, model for end-stage liver disease; MELD, model for end-stage liver disease.
